# No cancer predisposition or increased spontaneous mutation frequencies in NEIL DNA glycosylases-deficient mice

**DOI:** 10.1038/s41598-017-04472-4

**Published:** 2017-06-29

**Authors:** Veslemøy Rolseth, Luisa Luna, Ann Karin Olsen, Rajikala Suganthan, Katja Scheffler, Christine G. Neurauter, Ying Esbensen, Anna Kuśnierczyk, Gunn A. Hildrestrand, Anne Graupner, Jill M. Andersen, Geir Slupphaug, Arne Klungland, Hilde Nilsen, Magnar Bjørås

**Affiliations:** 1 Department of Microbiology, University of Oslo, Oslo University Hospital, Rikshospitalet, PO Box 4950 Nydalen, N-0424 Oslo, Norway; 2 Department of Clinical Molecular Biology, University of Oslo and Akershus University Hospital, Nordbyhagen, 1474 Norway; 3 Institute of Cancer Research and Molecular Medicine, Norwegian University of Science and Technology, 7491 Trondheim, Norway; 4 The Proteomics and Metabolomics Core Facility (PROMEC), Norwegian University of Science and Technology, 7491, Trondheim, Norway; 5 Centre of Environmental Radioactivity (CERAD CoE), Norway and Department of Molecular Biology, Norwegian Institute of Public Health, Oslo, Norway

## Abstract

Base excision repair (BER) is a major pathway for removal of DNA base lesions and maintenance of genomic stability, which is essential in cancer prevention. DNA glycosylases recognize and remove specific lesions in the first step of BER. The existence of a number of these enzymes with overlapping substrate specificities has been thought to be the reason why single knock-out models of individual DNA glycosylases are not cancer prone. In this work we have characterized DNA glycosylases NEIL1 and NEIL2 (*Neil1* ^−/−^ */Neil2* ^−/−^) double and NEIL1, NEIL2 and NEIL3 (*Neil1* ^−/−^ */Neil2* ^−/−^ */Neil3* ^−/−^) triple knock-out mouse models. Unexpectedly, our results show that these mice are not prone to cancer and have no elevated mutation frequencies under normal physiological conditions. Moreover, telomere length is not affected and there was no accumulation of oxidative DNA damage compared to wild-type mice. These results strengthen the hypothesis that the NEIL enzymes are not simply back-up enzymes for each other but enzymes that have distinct functions beyond canonical repair.

## Introduction

An inevitable consequence of aerobic metabolism is the production of reactive oxygen species (ROS)^1, 2^. In addition, exogenous sources such as cigarette smoking, pollutants, excessive alcohol consumption, asbestos, exposure to ionizing radiation, and bacterial, fungal or viral infections lead to enhanced generation of ROS^3–8^. ROS are well-described second messengers in a variety of cellular processes but ROS can be harmful at high concentrations becoming a threat to cells by causing peroxidation of lipids, oxidation of proteins, damage to nucleic acids and enzyme inhibition^9–14^. DNA lesions resulting from this type of damage are mutagenic and cytotoxic and, if not repaired, can cause genetic instability that may lead to various pathological conditions including neurodegenerative and cardiovascular diseases, carcinogenesis and premature aging^15–18^. The Base Excision Repair (BER) pathway is the primary mechanism responsible for the removal of small, non-helix-distorting base lesions that arise from oxidation as well as deamination and alkylation^19^. DNA glycosylases initiate BER by recognizing and excising the modified base, thereby forming an apurinic*/*apyrimidinic site (AP) site before repair is completed by the action of several enzymes that ultimately restore DNA integrity^19^.

Six mammalian DNA glycosylases initiate BER of a broad spectrum of oxidative DNA base lesions: *E. coli* Nei endonuclease VIII-like (NEIL) NEIL1, NEIL2 and NEIL3, 8-oxoguanine DNA glycosylase (OGG1), *Escherichia coli* (*E. coli*) MutY homologue (MUTYH) and *E. coli* Nth endonuclease III-like 1 (NTHL1)^19, 20^. 8*-*oxo-7,8*-*dihydroguanine (8-oxoG) is a major DNA oxidized lesion removed by OGG1^21, 22^. Further oxidation of 8-oxoG results in the formation of spiroiminodihydantoin (Sp) and guanidinohydantoin (Gh) that can both mispair with adenine and guanine^23–26^. The NEIL proteins have been shown to recognize and excise a number of oxidized bases including both Sp and Gh, from different substrates such as double- and single-stranded DNA, bubble DNA structures and telomeric and promoter quadruplex DNA structures^27–33^.

Thousands of endogenous DNA lesions are generated per day per cell making BER an essential pathway in preventing genomic damage and instability as seen in the numerous phenotypes observed in BER-deficient models. While deficiency of the common downstream enzymes of BER and the thymine-DNA glycosylase (TDG/Tdg) are embryonic lethal, the remaining DNA glycosylase-deficient mouse models have failed to display elevated mutation rates, increased cancer incidence or other severe phenotypes in the absence of any mutagenic insult^34–40^. This lack of altered phenotype exhibited by the single DNA glycosylase-deficient models has been ascribed to redundancy, or so-called back-up activities, between the enzymes as well as repair by alternative repair pathways.

In this paper, we describe the generation of single NEIL1 and NEIL2 (*Neil1* ^−/−^ and *Neil2* ^−/−^), NEIL1/NEIL2 double (*Neil1* ^−/−^ */Neil2* ^−/−^) and NEIL1/NEIL2/NEIL3 triple (*Neil1* ^−/−^ */Neil2* ^−/−^ */Neil3* ^−/−^) knock-out mouse models. We have used these models to study spontaneous tumour formation. Furthermore, the double and triple knock-out models have been used to determine oxidative damage accumulation in genomic DNA, relative telomere length and mutation frequencies in peripheral blood erythrocytes using the *in vivo Pig-a* gene mutation assay. Finally, we have derived primary mouse embryonic fibroblasts (MEFs) from our mouse models and determined cell survival after exposure to potassium dichromate.

## Results and Discussion

### Generation and characterization of *Neil1*^−/−^, *Neil2*^−/−^*, Neil1*^−/−^*/Neil2*^−/−^ and *Neil1*^−/−^*/Neil2*^−/−^*/Neil3*^−/−^ mice

**Figure 1:**
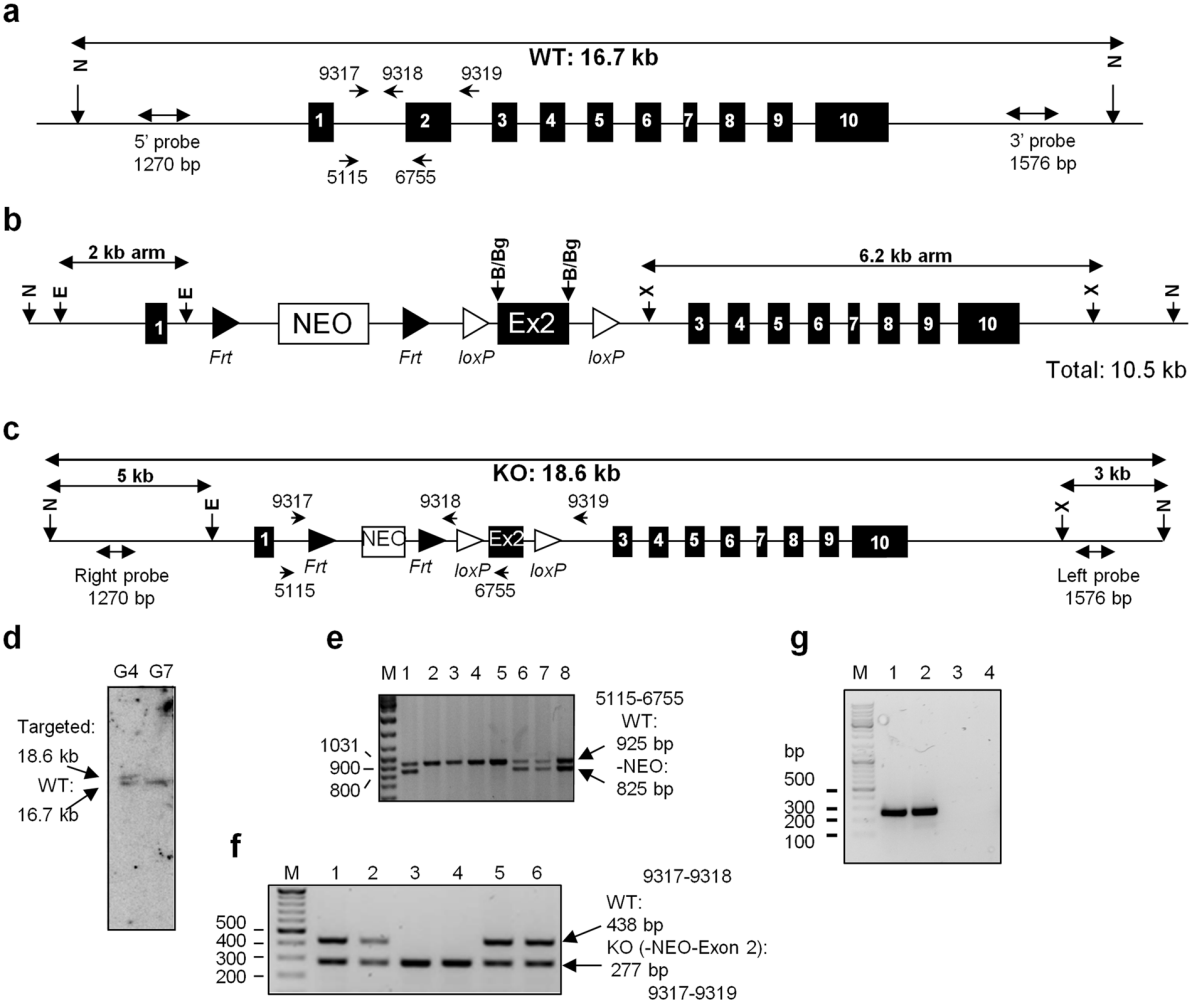
Targeted disruption of the murine *Neil1* locus by conditional knockout strategy. (**a**) The *Neil1* locus from the 129/SvJ mouse genome is shown. Exons, *NdeI* restriction enzyme sites, the position of the 5′ and 3′ probes used for Southern blotting and different primers used for genotyping during the generation of the *Neil1* ^−/−^ mice, are indicated. (**b**) The targeting construct carried two homology arms (2 kb and 6.2 kb), floxed exon 2 (Ex2) and an FRT-flanked neo cassette (NEO). (**c**) The targeted *Neil1* locus is shown. The positions of the 5′ and 3′ Southern blot probes are indicated. B and c The *loxP* sites are indicated by white triangles and the *Frt* sites are indicated by black triangles. Some restriction enzyme sites are shown; N: *NdeI*, E: *EcoRI*, B: *BamHI*, Bg: *BglII*, X: *XhoI*. (**d**) Southern blot analysis of DNA samples from two Geneticin-resistant ES clones digested with *NdeI* and hybridized with the 5′ probe identified a positive clone (G4). (**e**) Typical genotyping results obtained by PCR after breeding *Neil1* ^*+/floxneo*^ mice with FLP recombinase expressing mice to remove the FRT-flanked neo marker. (**f**) Typical genotyping results obtained by PCR after breeding *Neil1* ^+/*flox*^ mice with Cre recombinase expressing mice to remove the floxed exon 2 to generate *Nei1l* ^−/−^ mice. (**g**) Disruption of *Neil1* expression in *Nei1l* ^−/−^ mice was measured by RT-PCR analysis of mRNA isolated from brain. 1 and 2: WT; 3 and 4: *Nei1l* ^−/−^. (**e–g**) M: GeneRuler DNA ladder mix.

Conditional NEIL1 knock-out mice were generated by using the Cre-*loxP* recombination system. We made a gene-targeting vector in which the *loxP* sites flanked exon 2 of *Neil1* and a positive selection cassette for neomycin resistance flanked by *FRT* sequences (Fig. 1a and b). Exon 2 of NEIL1 contains the N terminal catalytic proline that forms a Schiff base with the oxidized lesion^41, 42^. The targeting construct was linearized with NdeI and electroporated into 129/SvJ embryonic stem cells (ESCs) to obtain the recombined *Neil1* locus (Fig. 1c). Genomic DNA was extracted from Geneticin-resistant ESC clones, NdeI*-*digested, and subjected to Southern blot analysis (Fig. 1d). One positive clone was identified (G4) and correct 5′ and 3′ targeting was confirmed by PCR. Recombinant ESCs derived from the G4 clone were injected into C57BL/6 blastocysts, which were transferred to pseudo-pregnant foster mothers (Norwegian Transgenic Center, Institute of Basic Medical Science, Faculty of Medicine, University of Oslo, Oslo, Norway). Chimeric male offspring were bred with C57BL/6N females to obtain *Neil1* ^+/*fl*^ *°* ^*xne*^ *°* mice. Cross-breeding with FLP recombinase-expressing mice was performed to remove the neomycin cassette (Fig. 1e). Cross-breeding with CMV-Cre-expressing mice was performed to remove the floxed exon 2 in all tissues (Fig. 1f). Inter-crossing of F_1_ *Neil1* ^+/−^ mice resulted in offspring of the expected Mendelian ratios. We examined *Neil1* expression levels by RT-PCR in brain tissue of *Neil1* ^+/+^ and *Neil1* ^−/−^ mice and as expected *Neil1* transcripts were absent in the knock-out mice (Fig. 1g). As previously observed by Vartanian and colleagues, *Neil1* ^−/−^ mice were viable and developed obesity late in life^36^.

**Figure 2:**
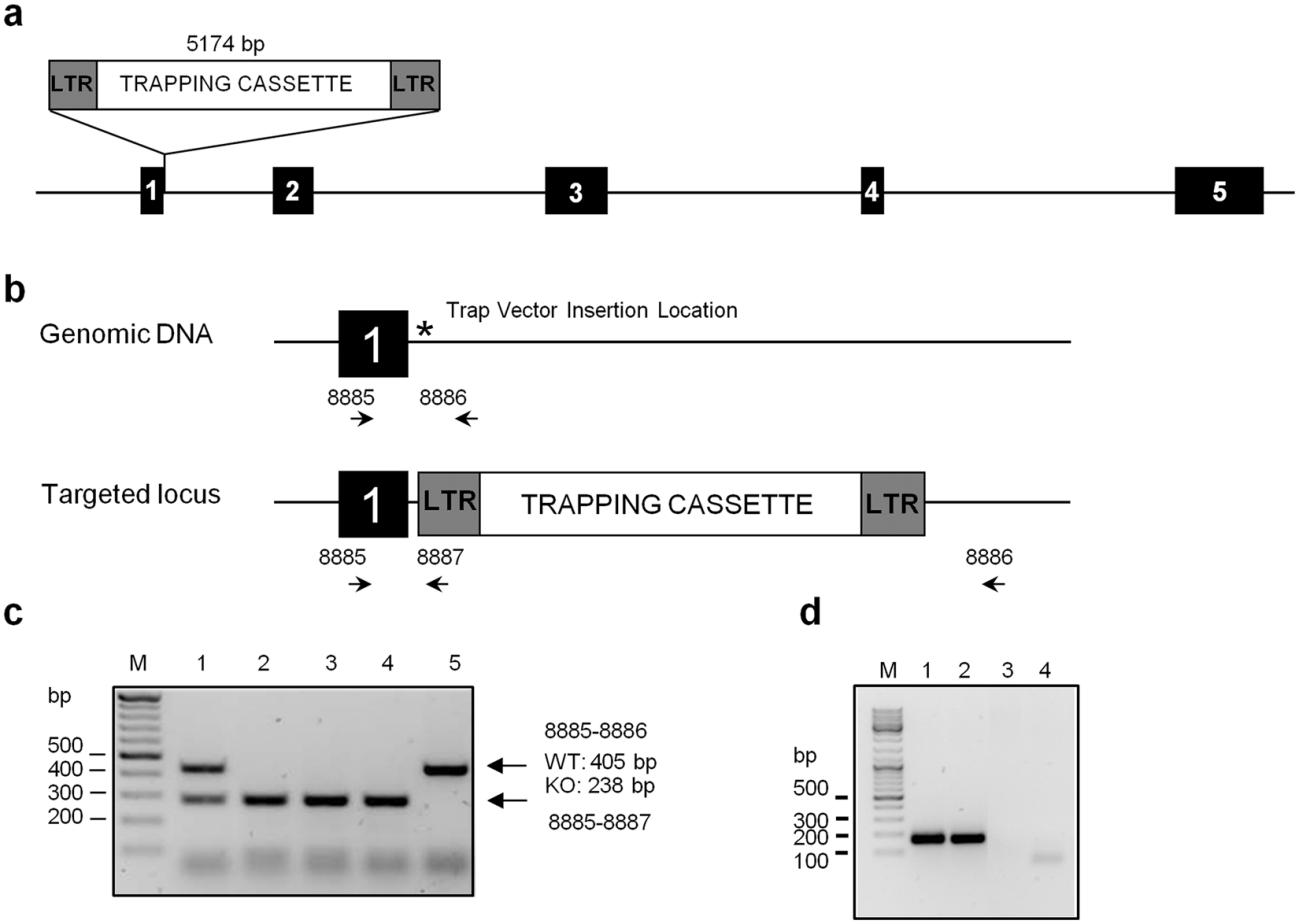
Targeted disruption of the murine *Neil2* locus by gene trapping. (**a**) The *Neil2* locus from the 129/SvJ mouse genome is shown with indicated exons. A retroviral gene trap vector was used to produce a clone: OST218883, which contains an insertion within the first intron of *Neil2*. This clone was used to generate *Neil2* ^−/−^ mice. (**b**) Detail of the *Neil2* locus from the 129/SvJ mouse genome and targeted *Neil2* locus showing the position of the primers used for genotyping. (**c**) Typical genotyping results obtained by PCR after breeding *Neil2* ^+/−^ mice with *Neil2* ^+/−^ mice. (**d**) Disruption of *Nei12* expression in *Neil2* ^−/−^ mice was measured by RT-PCR analysis of mRNA isolated from brain and thymus. 1: WT brain, 2: WT thymus, 3: *Neil2* ^−/−^ brain and 4: *Nei12* ^−/−^ thymus. (**c** and **d)** M: GeneRuler DNA ladder mix.

*Neil2* ^+/−^ mice were purchased from the Texas A&M Institute for Genomic Medicine, TX, USA. Briefly, *Neil2* ^+/−^ mice were generated using a 129/SvEv ES cell clone (Omnibank Clone OST218883) that contained a gene-trap vector insertion (Gene trapping vector VICTR48**)** within the first intron of *Neil2*. The insertion produced incorrect splicing, perturbing the expression of all *Neil2* downstream exons (Fig. 2a and b). *Neil2* ^−/−^ mice were generated by inter-crossing of F1 *Neil2* ^+/−^ mice. A PCR-based genotyping assay was established to distinguish wild-type and homozygous mice (Fig. 2c). Inter-crossing of *Neil2* ^+/−^ mice resulted in offspring of the expected Mendelian ratios. *Neil2* transcript levels were determined by RT-PCR in brain and thymus of *Nei2* ^+/+^ and *Neil2* ^−/−^ mice. No transcripts were detected in any of the organs tested (Fig. 2d). As observed by Chakraborty and co-workers^40^, the mice were viable and fertile and did not display any overt phenotype.

*Neil1* ^−/−^ */Neil2* ^−/−^ mice were generated by cross-breeding *Neil1* ^−/+^ */Neil2* ^+/+^ mice with *Neil1* ^+/+^ */Neil2* ^−/+^ mice (Supplementary Table 1). The resulting *Neil1* ^+/−^ */Neil2* ^+/−^ offspring was inter-crossed and genotyped to identify *Neil1* ^−/−^ */Neil2* ^−/−^ mice (Supplementary Fig. 1). *Neil1* ^−/−^ */Neil2* ^−/−^ */Neil3* ^−/−^ mice were generated by crossing *Neil1* ^−/−^ */Neil2* ^+/−^ */Neil3* ^+/+^ mice with *Neil1* ^+/+^ */Neil2* ^+/+^ */Neil3* ^−/−^ mice, generated previously in our lab^37^ (Supplementary Table 2). Offspring was cross-bred and genotyped until *Neil1* ^−/−^ */Neil2* ^−/−^ */Neil3* ^−/−^ mice were identified (Supplementary Fig. 1). Offspring of *Neil1* ^−/−^ */Neil2* ^−/−^ × *Neil1* ^−/−^ */Neil2* ^−/−^ and *Neil1* ^−/−^ */Neil2* ^−/−^ */Neil3* ^−/−^ × *Neil1* ^−/−^ */Neil2* ^−/−^ */Neil3* ^−/−^ breedings displayed no viability issues as indicated by normal weight gain and normal litter sizes as compared to the wild-type and single knock-out counterparts. The obese phenotype observed in *Neil1* ^−/−^ mice disappeared in the double and triple knock-out models. As for the single knock-outs, the double and triple knock-outs did not display any overt phenotype.

We looked for signs of stress such as loss of appetite and weight loss, lethargy, rough hair coat in colonies of wild-type, single, double and triple knock-outs. Mice that presented any of these signs were promptly euthanized and subjected to necropsy. We observed sporadic tumours in all strains including wild-type animals, however there was no overall difference in tumour formation between any of the knock-out models compared to the wild-type mice. Thus, none of the mouse models presented in this paper displayed increase in spontaneous tumour formation within 20 months. In previous DNA glycosylase-deficient mouse models such as OGG1 and MUTHY, the genetic background appeared to have an effect on the susceptibility to tumour formation^43–47^. However, our results are in agreement with previous studies of single NEIL1 and NEIL2 knock-out mice carried out in several laboratories^34, 36, 37, 40^.

Mice deficient in DNA glycosylases involved in removing oxidative bases were firstly developed to reveal the role of BER in tumorigenesis. Surprisingly, both NEIL1 and OGG1 deficient mice have been reported to develop late-onset obesity^36, 48, 49^. However, not all colonies of *Neil1* ^−/−^ or *Ogg1* ^−/−^ mice have displayed such a phenotype^50, 51^. Our *Neil1* ^−/−^ mice presented an obese phenotype that disappeared in the double and triple knock-out mice. At present we do not know the reason but due to the variability in penetrance of the phenotype, studies to address this would take many years and thousands of mice^48^. Some double knock-out mouse models like *Ogg1* ^−/−^ */Mutyh* ^−/−^ and *Nthl1* ^−/−^ */Neil1* ^−/−^ are predisposed to develop cancer, while others such as *Ogg1* ^−/−^ */Nthl1* ^−/−^ are not^46, 51, 52^. Interestingly, neither the double nor the triple knock-out models presented in this work displayed any sign of augmented spontaneous tumour formation. Accordingly, NTHL1 seems to play a role in removal of mutagenic pyrimidine lesions, while the nucleotide incision repair (NIR) can constitute repair activity against a subset of oxidized pyrimidines^53, 54^. Furthermore, repair activity towards hydantoins by the nucleotide excision repair (NER) in mammalian cells was recently reported^55^. Altogether, cells appear to be robustly equipped with a battery of mechanisms, to withstand normal physiological oxidative stress that could explain the lack of phenotype in our DNA glycosylase-deficient models.

The mice generated in the present work can now be crossed with other NER-deficient mouse models to assess the consequences of hydantoin accumulation and to evaluate the contribution of the different pathways to the repair of hydantoins in different tissues. Furthermore, the mice can be exposed to different oxidative insults to assess tumour formation.

### Normal telomere lengths in P0 and two year-old kidney, liver and spleen tissue

**Figure 3:**
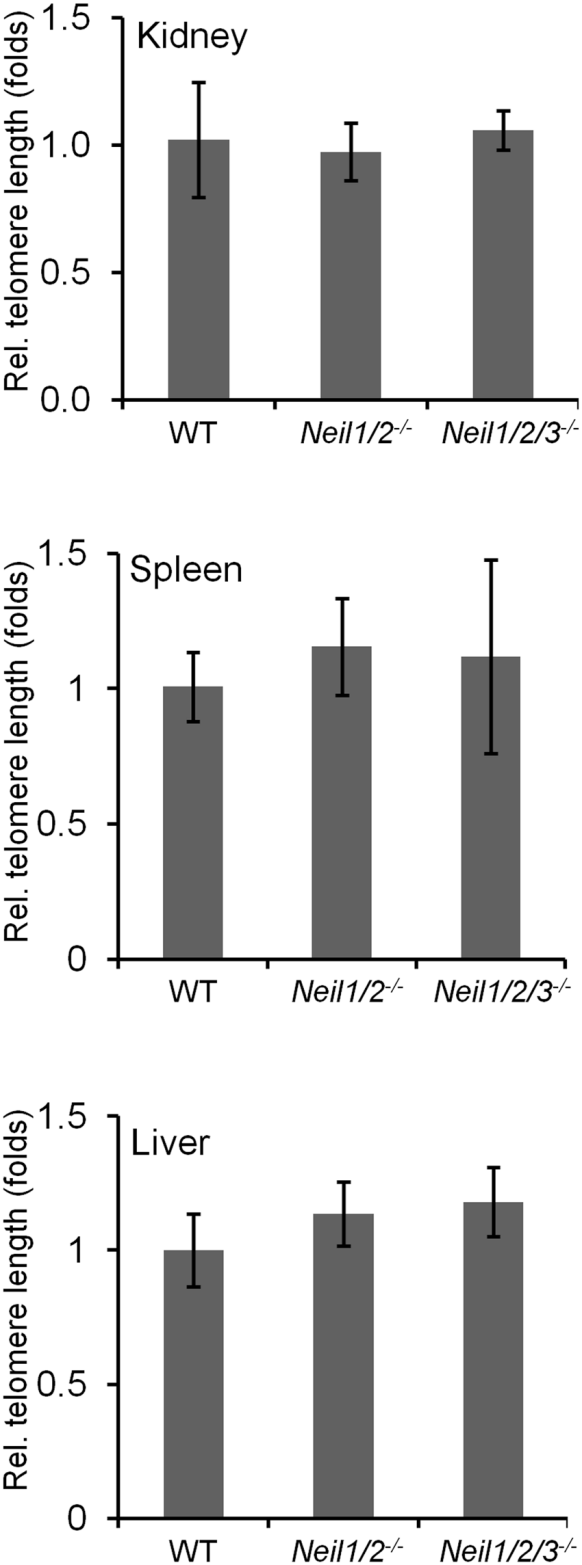
Quantitative analysis of telomere length. Relative telomere length was assessed in kidney, spleen and liver tissue isolated from three individual animals using qPCR in 2 year-old *Neil1* ^−/−^ */Neil2* ^−/−^ and *Neil1* ^−/−^ */Neil2* ^−/−^ */Neil3* ^−/−^ tissue compared to WT. Telomere length (mean ± SD) was presented relative to a single copy gene (36B4). *Neil1/2* ^−/−^: *Neil1* ^−/−^ */Neil2* ^−/−^; *Neil1/2/3* ^−/−^: *Neil1* ^−/−^ */Neil2* ^−/−^ */Neil3* ^−/−^.

Recent work has shown the likely involvement of the NEILs in both telomere maintenance and gene regulation^32, 33, 40^. Furthermore, telomere dysfunction has been shown to occur in NTHL1, OGG1, NEIL2 and UNG-deficient mouse models^40, 56–58^. We thus investigated spontaneous telomere length variation in liver, kidney and spleen from new-born and two year-old *Neil1* ^−/−^ */Neil2* ^−/−^ and *Neil1* ^−/−^ */Neil2* ^−/−^ */Neil3* ^−/−^ animals. As illustrated in Fig. 3 there was no significant change in the average telomere length in the double or triple knock-outs compared to wild-type in tissue harvested from two year-old mice. The same was true for tissue derived from new-born mice (data not shown).

### Organs from two year-old NEIL enzymes-deficient mice showed no increase in overall steady state levels of oxidized DNA base lesions

**Figure 4:**
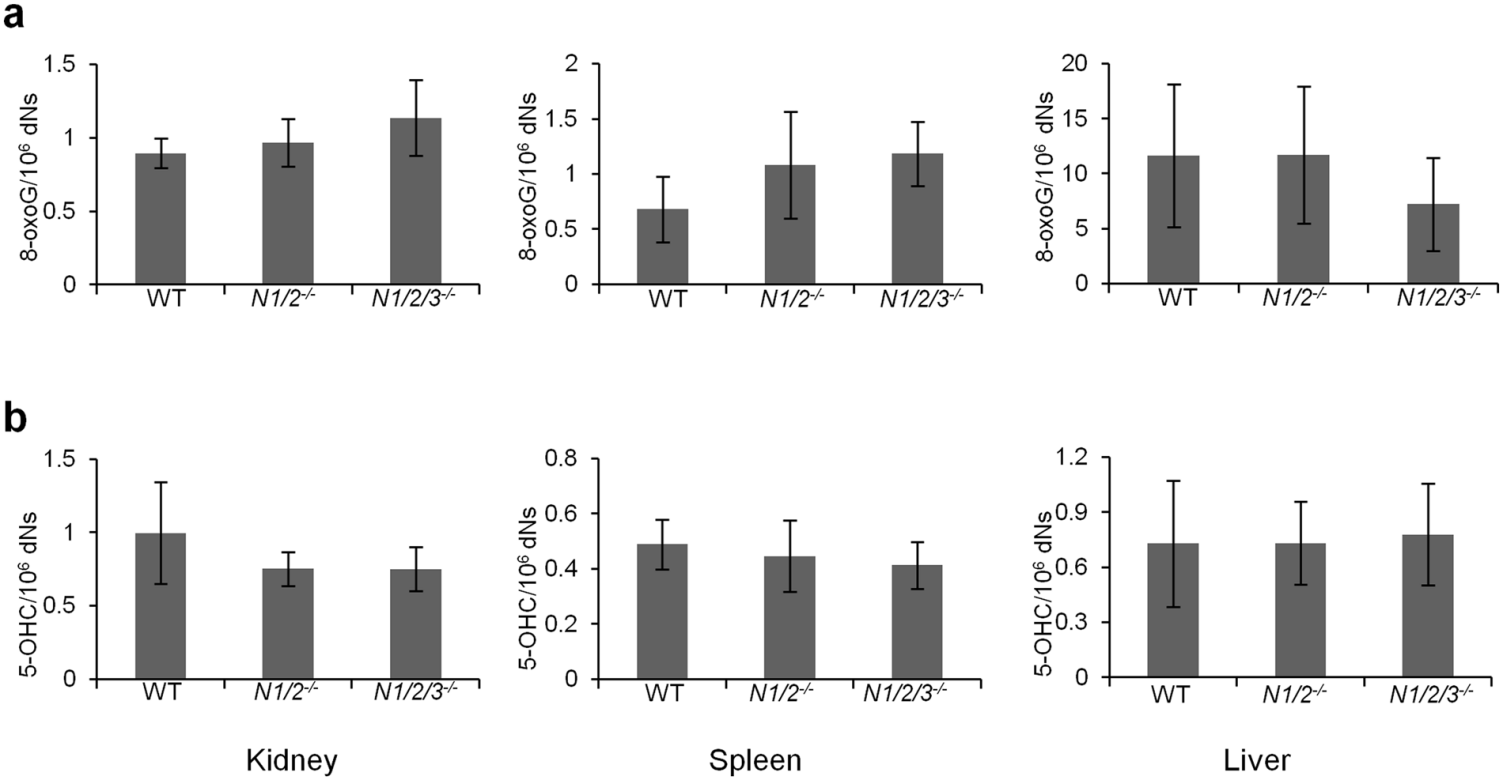
No accumulation of oxidized DNA bases. Oxidized DNA bases were measured in 2 year-old WT, *Neil1* ^−/−^ */Neil2* ^−/−^ and *Neil1* ^−/−^ */Neil2* ^−/−^ */Neil3* ^−/−^ tissue isolated from three individual animals. LC-MS/MS quantification of (**a**) 8-oxoG and (**b**) 5-OHC in kidney, spleen and liver. Data are presented as mean (±SD). *Neil1/2* ^−/−^: *Neil1* ^−/−^ */Neil2* ^−/−^; *Neil1/2/3* ^−/−^: *Neil1* ^−/−^ */Neil2* ^−/−^ */Neil3* ^−/−^. dNs: deoxyribonucleotides.

The NEIL proteins catalyze the excision of a broad range of oxidized bases^27–33^. We measured the levels of 8-oxoG and 5-OHC in genomic DNA derived from tissue isolated from two year-old animals. 5-OHC is a substrate for all the three NEIL enzymes whereas 8-oxoG is a good marker to indicate the extent of cellular oxidative stress. As seen in Fig. 4, the steady state levels of 8-oxoG and 5-OHC, in liver, kidney and spleen, did not differ between the two genotypes compared to wild-type. Sp and Gh, the secondary oxidation products of 8-oxoG have been detected both in *E. coli* and in murine liver and colon^59, 60^. However, the levels reported were nearly 100 times lower than 8-oxoG. We have tried to measure the *in vivo* levels of Sp and Gh in several tissues from our NEILs-deficient mice but failed so far. Our protocols are not sensitive enough.

### Spontaneous phenotypic mutation frequencies in *Neil1*^−/−^*/Neil2*^−/−^ and *Neil1*^−/−^*/Neil2*^−/−^*/Neil3*^−/−^ mice measured at the Pig-a gene

To elucidate whether the double or triple knock-out mouse models were associated with increased spontaneous phenotypic mutation frequencies we used the *Pig-a* gene mutation assay^61–65^. This assay is based on a cell surface phenotype characterized by the absence of membrane glycosylphosphatidylinositol (GPI)-anchored proteins such as CD24, CD48, CD59 and CD55^66^. Negative mutant hematopoietic cells were identified by fluorescence-activated cell sorting analysis. We measured mutation frequencies based on loss of a surface marker CD24 in immature RETs and mature RBCs. A definite advantage of the *Pig-a* gene mutation assay when compared to well-established mutagenicity tests like the HPRT or TK/MLA assay is the comparatively easy and fast procedure that interrogates high numbers of potential mutant cells (3–100 × 10^6^ cells/sample).

**Figure 5:**
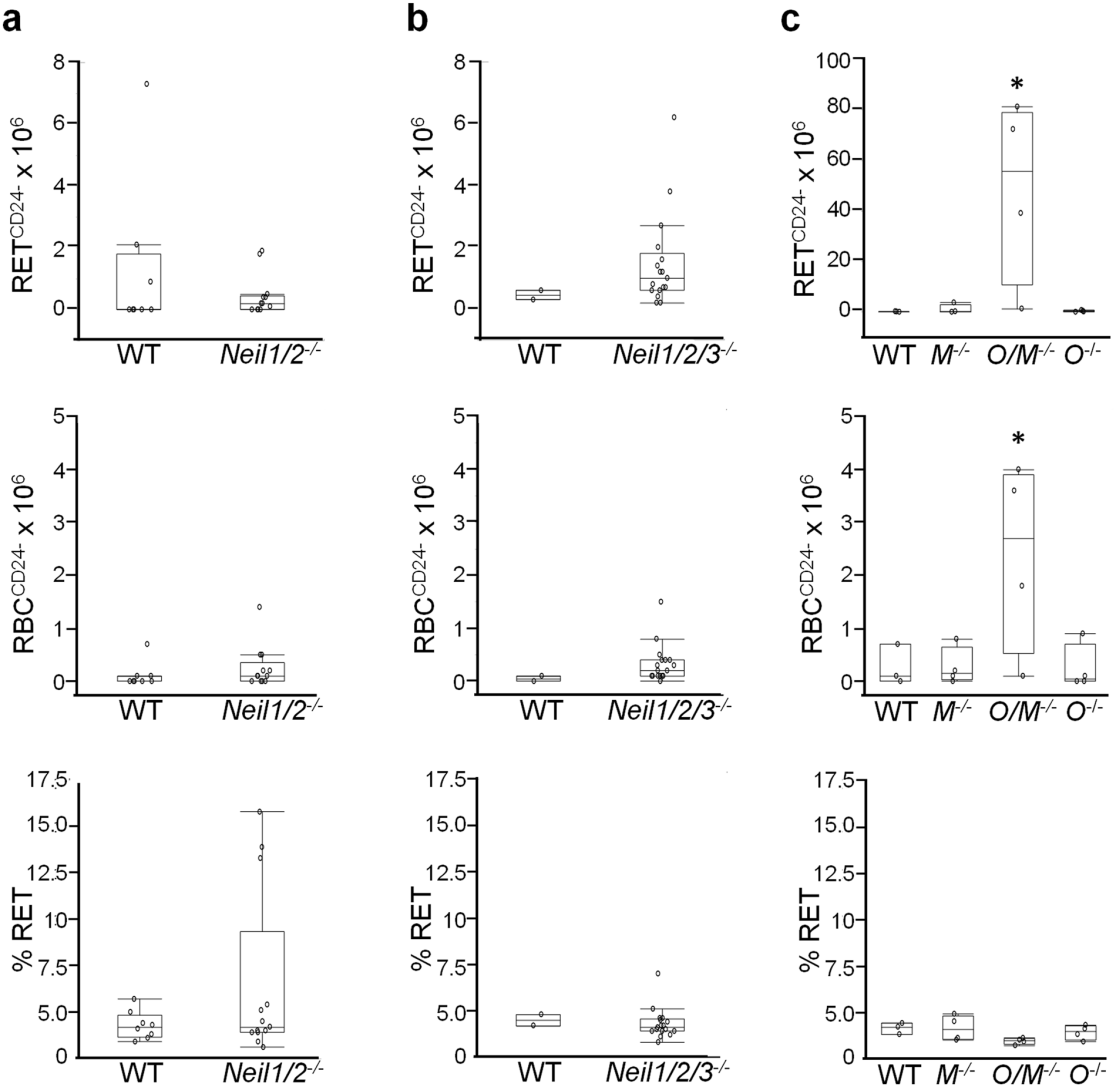
Phenotypic mutation frequencies measured by the *Pig-a* gene mutation assay. Frequencies of mutant phenotype reticulocytes (RET^CD24−^), erythrocytes (RBC^CD24−^) and percentage reticulocytes (% RET) were determined in (**a**) 18 months-old WT (n = 8) and *Neil1* ^−/−^ */Neil2* ^−/−^ (*Neil1/2* ^−/−^) (n = 13); (**b**) 23 months-old WT (n = 2) and *Neil1* ^−/−^ */Neil2* ^−/−^ */Neil3* ^−/−^ (*Neil1/2/3* ^−/−^) (n = 17) mice and **c)** 6 months-old WT (n = 3), *Mutyh* ^−/−^ (*M* ^−/−^) (n = 3), *Ogg1* ^−/−^ */Mutyh* ^−/−^ (*O/M* ^−/−^) (n = 3), and *Ogg1* ^−/−^ (*O* ^−/−^ *)* (n = 4) mice. Results are presented for three separate experiments **(a**–**c)**. Each data point represents one mouse; and groups are summarized as box-plots of at least two individuals. Groups that are significant different (p < 0.05) from control group (WT) are marked with an asterisk dependent on the distribution of the data, that is **(a)** non-parametric Wilcoxon test, **(b)** t-test and **(c)** Dunnett’s t﻿est.

Mammals posses three enzymes that prevent mutagenesis caused by 8-oxoG. OGG1 excises 8-oxoG from DNA, MUTYH removes adenine misincorporated opposite 8-oxoG in DNA and MTH1 (*mutT* homologue 1, NUDT1) degrades 8-oxodGTP in the nucleotide pool to prevent its incorporation into DNA^21, 22, 67, 68^. Since spontaneous and induced mutation frequencies in the single *Ogg1* ^−/−^and *Muthy* ^−/−^ and the double *Ogg1* ^−/−^ */Mutyh* ^−/−^ mouse models have been reported, we tested the sensitivity of the *Pig-a* gene mutation assay using 6 months-old single *Muthy* ^−/−^ and *Ogg1* ^−/−^ and double *Ogg1* ^−/−^ */Mutyh* ^−/−^ mice^43, 44, 46, 69–71^. I﻿ncreased mutagenic frequencies were detected﻿ in both RETs and in RBCs in the *Ogg1* ^−/−^ */Mutyh* ^−/−^ double knock-out mice compared to the wild-type mice, but not in the single *Ogg1* ^−/−^or *Muthy* ^−/−^ knock-out mice (Fig. 5c). To exclude the possibility that bone marrow cytotoxicity was the cause of the observed increase of spontaneous mutation frequencies, the percentage of RETs (% RET, ratio of newly formed RNA-positive erythrocytes relative to all erythrocytes) was calculated. As presented in Fig. 5c, lower panel, there was no significant change in the relative number of RETS in any of the mutants. Neither the 18 months-old *Neil1* ^−/−^ */Neil2* ^−/−^ mice nor the 23 months-old *Neil1* ^−/−^ */Neil2* ^−/−^ */Neil3* ^−/−^ mice displayed significantly increased *Pig-a* gene mutation frequencies (Fig. 5a and b).

It can be argued that the *Pig-a* gene mutation assay was not the best method to measure spontaneous mutation frequencies. However, its sensitivity is comparable to that of the Hprt assay and an increase in phenotypic mutation frequencies was detected in the *Ogg1* ^−/−^ */Mutyh* ^−/−^ double knock-out mice^72^. Altogether, the results presented endorse the *Pig-a* gene mutation assay as an *in vivo* mutation test that is sensitive, fast, reliable and less costly than other tests.

### Neither hydrogen peroxide nor potassium dichromate treatment affect the survival of NEILs-deficient MEF cells

**Figure 6:**
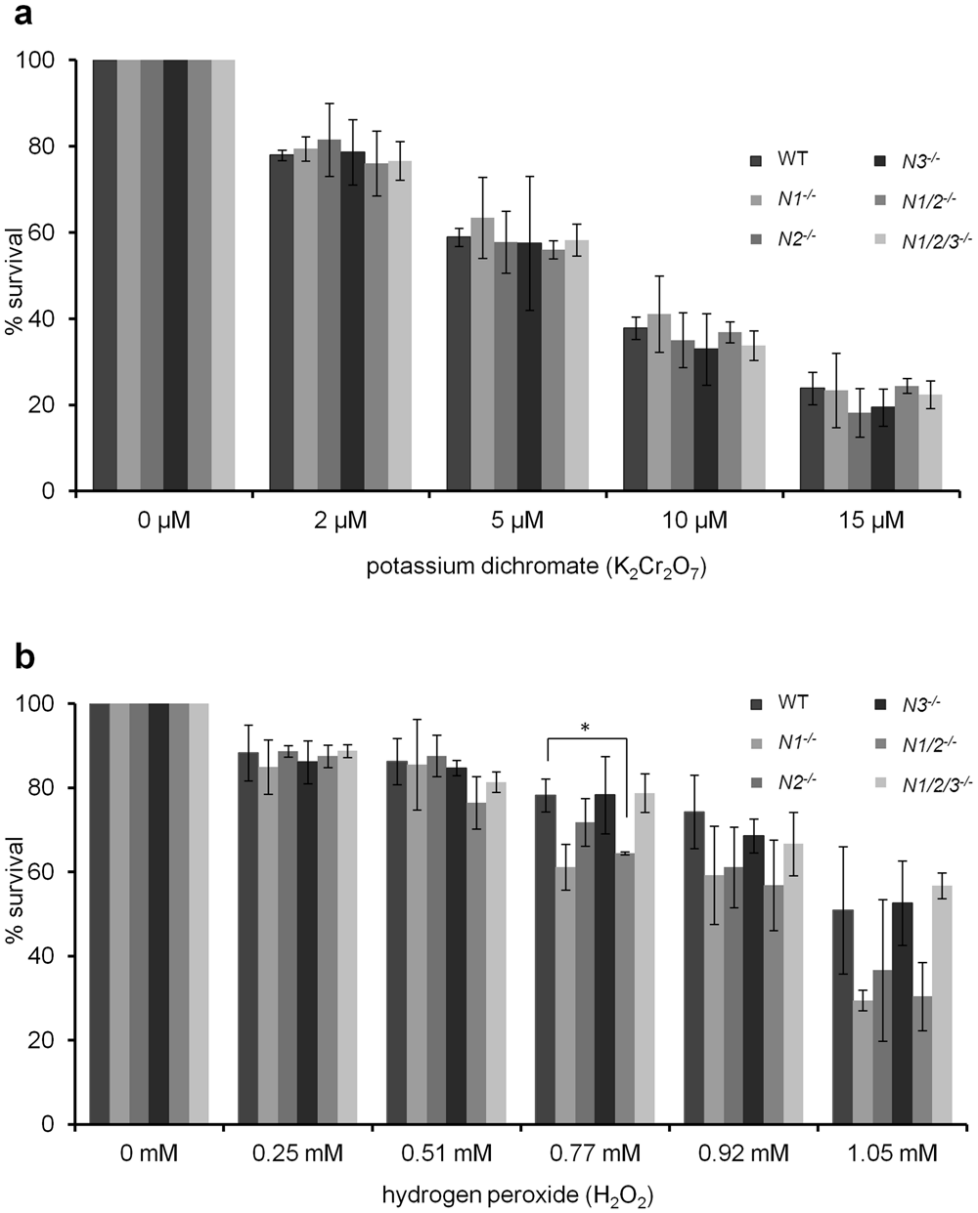
Primary MEFs survival response to hydrogen peroxide and potassium dichromate. Cell survival response towards increasing concentrations of (**a**) potassium dichromate and (**b**) hydrogen peroxide was measured using the MTT assay after 24 hour exposure. MEF cells: *N1* ^−/−^: *Neil1* ^−/−^, *N2* ^−/−^: *Neil2* ^−/−^: *N3* ^−/−^: *Neil3* ^−/−^, *Neil1/2* ^−/−^: *Neil1* ^−/−^ */Neil2* ^−/−^, *Neil1/2/3* ^−/−^: *Neil1* ^−/−^ */Neil2* ^−/−^ */Neil3* ^−/−^. Results are mean (±SD) OD (% of control) of three to five independent experiments for each genotype. *p < 0.005 compared to WT.

Because we did not observe any phenotype under normal conditions, we wanted to investigate if exposure of MEFs to DNA damaging agents such as hydrogen peroxide or potassium dichromate had any consequences. Further oxidation of 8-oxoG by for example high valent metals such as chromium (V) results in the generation of the lesions Sp and Gh. In *E. coli*, these lesions are repaired by both Fpg and Nei and in mammalian cells by the NEIL proteins and NTHL1. Moreover, it has been shown that Nei-deficient *E. coli* are sensitive to chromate exposure^59^. Cells were exposed to increasing amounts of potassium dichromate and hydrogen peroxide for 24 hours and cell viability was measured using the MTT assay. As shown in Fig. 6a none of the DNA glycosylase-deficient MEFs were more sensitive to potassium dichromate than wild-type MEF cells. We observed significant sensitivity of *Neil1* ^−/−^ */Neil2* ^−/−^ MEFs compared to wild-type towards hydrogen peroxide at only one concentration (Fig. 6b). However, none of the other MEFs were significantly more sensitive compared to wild-type cells. A narrow window of vulnerability cannot be ruled out, but in general, NEIL1, NEIL2 and/or NEIL3 deficiency is not enough to render MEF cells sensitive towards hydrogen peroxide or potassium dichromate. This lack of sensitivity is most likely due to the removal of oxidized lesions including Sp and Gh by other DNA glycosylases such as NTHL1 and by other pathways. NER and DNA mismatch repair (MMR) have also been shown to have a role in the repair of oxidative DNA damage^73, 74^. More recently, NER has been shown to mediate repair of hydantoin lesions in cells^55^.

## Conclusions

The results presented in this paper strongly indicate that the NEIL DNA glycosylases are not back-up enzymes for each other in processing of oxidized DNA bases. NEIL1 has been implicated in the repair of base lesions during replication^75–77^, whereas NEIL2 has been suggested to be relevant for transcription associated BER^40, 78^. NEIL3-dependent repair of oxidative DNA damage appears to be important in proliferating cells, including neural stem/progenitor cells required for induced and continuous neurogenesis^37, 79^. Altogether it is plausible to suggest that NEIL1, NEIL2 and NEIL3 DNA glycosylases have evolved to carry out different specialized functions that do not overlap. We hypothesize that the enzymes carry out specific functions that are coordinated or associated with cellular processes such as cell cycle progression, transcription, replication and chromatin remodelling.

## Methods

### Ethics statement

This study was carried out in accordance with the Norwegian Regulation on Animal Experimentation, which is based upon the European Convention for the Protection of Vertebrate Animals Used for Experimental and Other Scientific Purposes. All experiments were approved by the Norwegian Animal Research Committee.

### Reagents

Lympholyte®-Mammal cell separation reagent was purchased from CedarLane (Burlington, ON, Canada). Anti-PE MicroBeads, LS + Positive Selection Columns and a QuadroMACS™ Separator were from Miltenyi Biotec (GmbH, Bergisch Gladbach, Germany). CountBright™ Absolute Counting Beads were from Invitrogen, Life Technologies™ (Carlsbad, CA, USA). Heat-inactivated fetal bovine serum (FCS) was from PAA Laboratories (Pasching, Austria). DMEM medium, trypsin/EDTA solution, penicillin/streptomycin was from BioWhittaker™ (Walkersville, MD, USA). GlutaMAX™ Supplement, was from Gibco (Grand Island, NY, USA). Anticoagulant solution, buffered salt solution, nucleic acid dye solution (SYTO^®^13), anti-CD24-PE and anti-CD61-PE were from the Prototype *In Vivo* Mouse MutaFlow^®^ Kit, Litron Laboratories (Rochester, NY, USA). N-ethyl-N-nitrosourea (ENU, CAS 759–73–9, cat. no. N3385), potassium dichromate (cat. no. 207802), Hydrogen peroxide solution 30 wt. % in H_2_O (cat. no. 216763) and DMSO were from Sigma Aldrich (St. Louis, MO, USA). Cell proliferation kit (MTT) (cat. no. 11465007001) was from Roche Diagnostic (Roche Inc., Mannheim, Germany).

### Primers

Primers used for genotyping and RT-PCR are listed in Table 1.

**Table 1:** Primers used for genotyping and RT-PCR.

Target	Sequence (5′-3′)
Genotyping	
*Neil1*	
9317	AGGTGCTAGAACAGATGCTGAGT
9318	CCAGAAATGTCTTGTTCAGTAGGA
*9319*	CGATCCGGAACCCTTAATATAAC
*Neil2*	
8885	CCTTCGGAAGGGTTAGGAAACG
8886	TACTCTGACCTTCAGGGAAAGC
8887	ATAAACCCTCTTGCAGTTGCATC
*Neil3*	
For	GCCTCTGTTCCACATACACTTCAT
WT-Rev	CTTGTTTTCCCACCACAATCTG
KO-Rev	GTGGGCTGAAATTACACAAACAAT
RT-PCR	
*Neil1*	CGCCTGCCTGAGAAAACTAC
	CACACACCCACCAAATACCA
*Neil2*	CGCTGTGGCCTAAGACTTTC
	AAAGGGGGAGACAAGATGGT

### Animals

All strains were back-crossed more than 8 generations onto a C57BL/6N background (N8 = 99.6%). A total of 55 animals were used for the *Pig-a* gene mutation experiments: 3 wild-type (WT), 3 *Mutyh* ^−/−^, 4 *Ogg1* ^−/−^ and 3 *Ogg1* ^−/−^ */Mutyh* ^−/−^ 6 months-old; 8 WT and 13 *Neil1* ^−/−^ */Neil2* ^−/−^ 18 months-old and 2 WT and 17 *Neil1* ^−/−^ */Neil2* ^−/−^ */Neil3* ^−/−^ 23 months-old mice. For a positive control of the *Pig-a* gene mutation assay, 2 WT mice were treated with ENU (i.p. injection, 40 mg/kg bodyweight (bw) on three consecutive days, total dose of 120 mg/kg bw).

### Longevity studies

Mice were allowed to age and were observed for development of disease and subject to full necropsy at age between 18 and 24 months-old.

### Primary mouse embryonic fibroblasts and exposure experiments

Primary mouse embryonic fibroblasts (MEFs) were generated from E14.5-E-15.5 mouse tissue of WT, *Neil1* ^−/−^, *Neil2* ^−/−^, *Neil3* ^−/−^, *Neil1* ^−/−^ */Neil2* ^−/−^ and *Neil1* ^−/−^ */Neil2* ^−/−^ */Neil3* ^−/−^ embryos. Limbs were removed from embryos, the tissue was chopped into small pieces and cell suspension was made using a pipette. MEFs were grown in DMEM supplemented with 10% serum, 2 mM glutamine and penicillin/streptomycin. Cells grown for 3–4 days were trypsinized and frozen in complete medium with 10% DMSO as stock. After thawing, cells were grown in complete medium for 2–4 days before using them for survival assays. Primary MEFs were seeded in 96 well plates at a concentration of either 4000 or 8000 cells/well, incubated for 4 h and then treated with increasing amounts of potassium dichromate (K_2_Cr_2_O_7_) or hydrogen peroxide (H_2_O_2_) for 24 h. Results were calculated from at least three independent experiments carried out in quadruplicate. Cell viability was determined with the MTT Cell Proliferation kit (Roche Diagnostics) and absorbance was measured on a Wallac 1420 Multilabel Counter at a wavelength of 570 nm and reference wavelength 670 nm. Cell viability was calculated as percentage relative to control cells set as 100%.

### Mutation frequencies at the Pig-a gene

Blood samples were taken from the saphenous vein using a 21-G needle and heparinised capillary tube. 60 µl of free-flowing blood were added to 100 µl anticoagulant, mixed well and kept at room temperature until further processing within two hours. The *Pig-a* gene mutation assay was performed as described previously^61–63^ and in the Prototype Mouse MutaFlow^®^ Kit instruction manual (version120301). At least 100 × 10^6^ red blood cells (RBC) and 3 × 10^6^ reticulocytes (RET) per sample were recorded. The Prototype MutaFlow® protocol used herein included a post-column incubation step of 30 min at 37 °C, a step that was discovered to potentially lyse mouse erythrocytes, especially RNA-negative erythrocytes. This implies that the RBC^CD24−^ values presented herein may be somewhat underestimated.

The data for *Pig-a* gene mutation assay was processed and calculated as described in ref. 80 using Microsoft Excel 2010. Data was log(10) transformed (an offset of 0.1 was added for RET^CD24−^ and RBC^CD24−^ values prior transformation to avoid zero) and further statistics was performed depending on the distribution using JMP Pro 12 (Statistical Analysis System Institute Inc., Cary, NC, USA). That is, t-test for data from 23 months-old *Neil1* ^−/−^ */Neil2* ^−/−^ */Neil3* ^−/−^ mice; non-parametric Wilcoxon test for 18 months-old *Neil1* ^−/−^ */Neil2* ^−/−^ mice and Dunnett’s for 6 months-old *Ogg1* ^−/−^ */Mutyh* ^−/−^ mice.

### DNA extraction

DNA was isolated from P0 and two year-old tissues using DNeasy Blood and Tissue Kit (Qiagen, 69506), according to the manufacturer’s protocol.

### Telomere relative length quantification

Relative telomere length was determined by qPCR as described^81^. Briefly, after quantifying genomic DNA extracted from each sample, real-time SYBR Green qPCR was performed on a Step One Plus Real-Time PCR system (Applied Biosystems) using two pairs of primers, one telomere specific and one single copy gene (36B4). Relative telomere relative length quantification was determined using the relative standard curve method. Each sample was loaded in triplicate.

### LC-MS/MS analysis of 8-oxoG and 5-hydroxycytosine in genomic DNA

Two µg of genomic DNA was enzymatically hydrolyzed to deoxyribonucleosides by incubation in a mixture of benzonase (sc-391121B, Santa Cruz Biotechnology, Heidelberg, Germany) nuclease P1 from *Penicillium citrinum* (N8630, Sigma Aldrich, St. Louis, MO, USA) and alkaline phosphatase from *E. coli* (P5931, Sigma Aldrich, St. Louis, MO, USA) in 10 mM ammonium acetate buffer, pH 5.3 at 40 °C for 40 min. Three volume equivalents of ice-cold methanol were added to the reactions after digestion was completed to precipitate proteinaceous contaminants. Following centrifugation at 16000 × *g* at 4 °C for 40 min the supernatants were collected in new tubes and dried under vacuum at room temperature. The resulting residues were dissolved in 50 µl of water for LC-MS/MS quantification of 8-oxoG and 5-hydroxydeoxycytosine (5-OHC). For quantification of unmodified nucleosides (dA, dC, dG, dT) samples were further diluted with water up to 1:5000. Chromatographic separation of nucleosides was performed using a Shimadzu Prominence LC-20AD HPLC system with an Ascentis Express C18 2.7 µm 150 × 2.1 mm i.d. column equipped with an Ascentis Express Cartridge Guard Column (Supelco Analytical, Bellefonte, PA, USA) with EXP Titanium Hybrid Ferrule (Optimize Technologies Inc.) at a flow rate of 0.14 ml/min at ambient temperature. The mobile phase consisted of A (0.1% formic acid in water) and B (0.1% formic acid in methanol). The following conditions were employed during chromatographic separation: unmodified nucleosides –starting with 90% A and 10% B for 0.1 min, followed by a 2.4 min linear gradient of 10–60% B, and 4.5 min re-equilibration with the initial mobile phase conditions; 8-oxoG and 5-OHC – starting with 95% A and 5% B for 0.5 min, followed by a 7.5 min linear gradient of 5–45% B, and 5.5 min re-equilibration with the initial mobile phase conditions. Online mass spectrometry detection was performed using an Applied Biosystems/MDS Sciex API5000 Triple quadrupole mass spectrometer (ABsciex, Toronto, Canada), operating in positive electrospray ionization mode. The deoxyribonucleosides were monitored by multiple reaction monitoring using mass transitions 252.2 → 136.1 (dA), 228.2 → 112.1 (dC), 268.2 → 152.1 (dG), 243.2 → 127.0 (dT), 284.1 → 168.1 (8-oxoG) and 244.1 → 128.0 (5-OHC).

## Electronic supplementary material

Supplementary Info
