# Supplementary material for: No cancer predisposition or increased spontaneous mutation frequencies in NEIL DNA glycosylases-deficient mice
Source: Sci Rep. 2017 Jun 29;7:4384. doi: 10.1038/s41598-017-04472-4 (PMC5491499; doi:10.1038/s41598-017-04472-4)
Supplement: Supplementary file 1 — Supplementary Info [file 41598_2017_4472_MOESM1_ESM.pdf]

No cancer predisposition or increased spontaneous mutation frequencies in NEIL DNA glycosylases-deficient mice.

Veslemøy Rolseth[1#*], Luisa Luna[1#*], Ann Karin Olsen[5], Rajikala Suganthan[1], Katja Scheffler[3], Christine G Neurauter[1], Ying Esbensen[2], Anna Kuśnierczyk[3, 4], Gunn A Hildrestrand[1], Anne Graupner[5], Jill M. Andersen[5], Geir Slupphaug[3, 4], Arne Klungland[1], Hilde Nilsen[2] and Magnar Bjørås[1,3*]

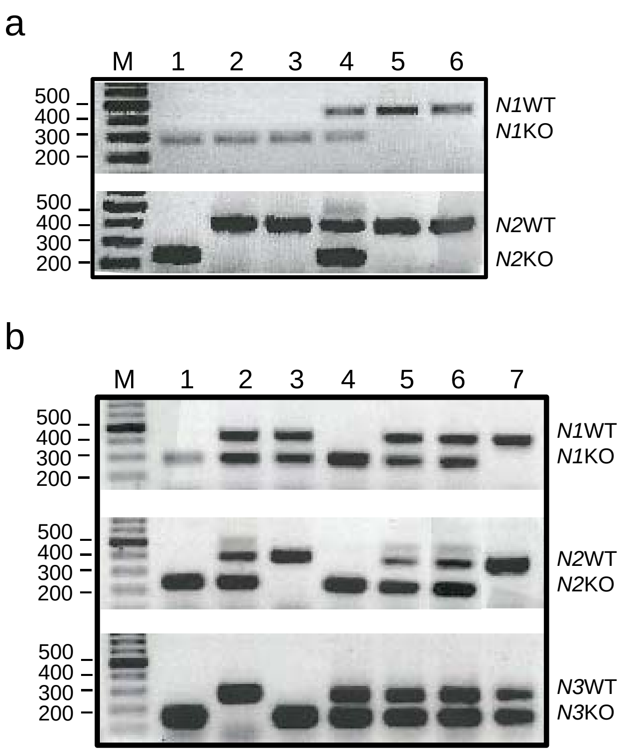

Supplementary Fig. 1

Typical genotyping results obtained by PCR after cross-breeding:

a) Genotyping of *Neil1*$^{-/-}$ with *Neil2*$^{-/-}$ mice offspring. Upper panel are *Neil1* primers, lower panel are *Neil2* primers. Sample 1 represents *Neil1*$^{-/-}$/*Neil2*$^{-/-}$; samples 2 and 3 represent *Neil1*$^{-/-}$/*Neil2*$^{+/+}$; sample 4 represents *Neil1*$^{+/-}$/*Neil2*$^{+/-}$ and samples 5 and 6 represent *Neil*$^{+/+}$/*Neil2*$^{+/+}$. **b)** Genotyping of *Neil1*$^{-/-}$/*Neil2*$^{-/-}$ with *Neil3*$^{-/-}$ mice offspring. Upper panel are *Neil1* primers, middle panel are *Neil2* primers and lower panel are *Neil3* primers. Sample 1 represents *Neil1*$^{-/-}$/*Neil2*$^{-/-}$/*Neil3*$^{-/-}$; sample 2 represents *Neil1*$^{+/-}$/*Neil2*$^{+/-}$/*Neil3*$^{+/+}$; sample 3 represents *Neil1*$^{+/-}$/*Neil2*$^{+/+}$/*Neil3*$^{-/-}$; sample 4 represents *Neil1*$^{-/-}$/*Neil2*$^{-/-}$/*Neil3*$^{+/-}$; samples 5 and 6 represent *Neil1*$^{+/-}$/*Neil2*$^{+/-}$/*Neil3*$^{+/-}$ and sample 7 represents *Neil1*$^{+/+}$/*Neil2*$^{+/+}$/*Neil3*$^{+/-}$. **a and b)** M: GeneRuler DNA ladder mix.

Table S1: Crossings to generate *Neil1$^{-/-}$/Neil2$^{-/-}$* mice

Neil1$^{-/+}$/Neil2$^{+/+}$* x *Neil1$^{+/+}$/Neil2$^{-/+}$	**Genotype**	**Sex**	**Nr.**	**Litter size**
	Neil1/Neil2			
♀ *Neil1$^{-/+}$/Neil2$^{+/+}$* x ♂ *Neil1$^{+/+}$/Neil2$^{-/+}$*	Het/WT	♀	1	6
	Het/Het	♀	2	
	Het/Het	♀	3	
	Het/Het	♀	4	
	WT/Het	♂	5	
	Het/Het	♀	6	
♀ *Neil1$^{-/+}$/Neil2$^{+/+}$* x ♂ *Neil1$^{+/+}$/Neil2$^{-/+}$*	Het/Het	♀	7	8
	WT/WT	♀	8	
	Het/Het	♀	9	
	WT/Het	♀	10	
	Het/WT	♂	11	
	WT/WT	♂	12	
	WT/Het	♂	13	
	Het/Het	♂	14	
♂ *Neil1$^{-/+}$/Neil2$^{+/+}$* x ♀ *Neil1$^{+/+}$/Neil2$^{-/+}$*	WT/Het	♀	15	7
	Het/WT	♀	16	
	Het/Het	♀	17	
	Het/Het	♀	18	
	WT/Het	♂	19	
	Het/WT	♂	29	
	Het/Het	♂	30	
♂ *Neil1$^{-/+}$/Neil2$^{+/+}$* x ♀ *Neil1$^{+/+}$/Neil2$^{-/+}$*	Het/Het	♀	31	8
	Het/Het	♀	32	
	Het/WT	♀	33	
	Het/Het	♀	34	
	WT/WT	♀	35	
	WT/WT	♀	36	
	Het/WT	♀	37	
	WT/WT	♂	38	

Neil1$^{-/+}$/Neil2$^{+/+}$* x *Neil1$^{+/+}$/Neil2$^{-/+}$

	Expected %	Observed	Observed %	♀	♂
WT/WT	25	5/29	17	3	2
WT/Het	25	5/29	17	2	3
Het/WT	25	6/29	21	4	2
Het/Het	25	13/29	45	11	2

Neil1$^{-/+}$/Neil2$^{-/+}$* x *Neil1$^{-/+}$/Neil2$^{-/+}$	**Genotype**	**Sex**	**Nr.**	**Litter size**
	Neil1/Neil2			
♀ *Neil1$^{-/+}$/Neil2$^{-/+}$* x ♂ *Neil1$^{-/+}$/Neil2$^{-/+}$*	WT/Het	♀	1	5
	Het/WT	♀	2	
	Het/Het	♀	3	
	**KO/KO**	♂	4	
	Het/Het	♂	5	
♀ *Neil1$^{-/+}$/Neil2$^{-/+}$* x ♂ *Neil1$^{-/+}$/Neil2$^{-/+}$*	**KO/KO**	♀	6	7
	Het/KO	♀	7	
	Het/Het	♀	8	
	Het/WT	♂	9	
	KO/WT	♂	10	
	Het/WT	♂	11	
	Het/KO	♂	12	
♀ *Neil1$^{-/+}$/Neil2$^{-/+}$* x ♂ *Neil1$^{-/+}$/Neil2$^{-/+}$*	Het/Het	♀	13	9
	KO/WT	♀	14	
	KO/WT	♀	15	
	KO/Het	♀	16	
	WT/Het	♀	17	
	KO/Het	♂	18	
	Het/Het	♂	19	
	Het/KO	♂	20	
	Het/Het	♂	21	
♀ *Neil1$^{-/+}$/Neil2$^{-/+}$* x ♂ *Neil1$^{-/+}$/Neil2$^{-/+}$*	KO/Het	♂	22	6
	Het/Het	♂	23	
	KO/Het	♂	24	
	WT/KO	♂	25	
	KO/Het	♂	26	
	Het/Het	♂	27	

Neil1$^{-/+}$/Neil2$^{-/+}$* x *Neil1$^{-/+}$/Neil2$^{-/+}$

	Expected %	Observed	Observed %	♀	♂
WT/WT	6.3	5/56	8.9	2	3
WT/Het	12.5	3/56	5.3	2	1
WT/KO	6.3	3/56	5.3	2	1
Het/WT	12.5	5/56	8.9	2	3
Het/Het	25	16/56	28.6	7	9
Het/KO	12.5	5/56	8.9	2	3
KO/WT	6.3	4/56	7.1	2	2
KO/Het	12.5	10/56	17.9	3	7
KO/KO	6.3	5/56	8.9	4	1

♀ Neil1$^{-/+}$/Neil2$^{-/+}$ x ♂ Neil1$^{-/+}$/Neil2$^{-/+}$	WT/WT	♀	28	6
	Het/WT	♂	29	
	KO/Het	♂	30	
	Het/KO	♂	31	
	Het/Het	♀	32	
	KO/Het	♀	33	
♀ Neil1$^{-/+}$/Neil2$^{-/+}$ x ♂ Neil1$^{-/+}$/Neil2$^{-/+}$	Het/Het	♀	34	7
	KO/Het	♀	35	
	Het/Het	♂	36	
	KO/Het	♂	37	
	KO/Het	♂	38	
	WT/WT	♂	39	
	Het/Het	♂	40	
♀ Neil1$^{-/+}$/Neil2$^{-/+}$ x ♂ Neil1$^{-/+}$/Neil2$^{-/+}$	Het/Het	♀	41	9
	**KO/KO**	♀	42	
	WT/KO	♀	43	
	WT/KO	♀	44	
	Het/Het	♀	45	
	WT/WT	♀	46	
	WT/WT	♂	47	
	KO/WT	♂	48	
	WT/Het	♂	49	
♀ Neil1$^{-/+}$/Neil2$^{-/+}$ x ♂ Neil1$^{-/+}$/Neil2$^{-/+}$	**KO/KO**	♀	50	7
	**KO/KO**	♀	51	
	Het/KO	♀	52	
	Het/WT	♀	53	
	Het/Het	♂	54	
	WT/WT	♂	55	
	Het/Het	♂	56	

Pairings: ***Neil1$^{-/-}$/Neil2$^{-/-}$ x Neil1$^{-/-}$/Neil2$^{-/-}$***

Litter size	♀	♂
9	1	8
7	6	1
8	4	4
9	5	4
9	4	5
5	2	3
8	3	5
11	5	6
5	1	4
7	3	4
6	3	3
9	6	3
8	5	3
9	5	4
7	1	6
8	6	2

Genotype	Sex	Total number
KO/KO	♀	60
KO/KO	♂	65

Table S2: Crossings to generate *Neil1$^{-/-}$/Neil2$^{-/-}$/Neil3$^{-/-}$* mice

Neil1$^{+/+}$/Neil2$^{+/+}$/Neil3$^{-/-}$ x *Neil1$^{-/-}$/Neil2$^{+/-}$/Neil3$^{+/+}$*	Genotype	Sex	Nr.	Litter size
	Neil1/Neil2/Neil3			
♀ *Neil1$^{+/+}$/Neil2$^{+/+}$/Neil3$^{-/-}$* x ♂ *Neil1$^{-/-}$/Neil2$^{+/-}$/Neil3$^{+/+}$*	Het/Het/Het	♂	1	7
	Het/WT/Het	♂	2	
	Het/WT/Het	♂	3	
	Het/Het/Het	♂	4	
	Het/WT/Het	♂	5	
	Het/Het/Het	♀	6	
	Het/WT/Het	♀	7	
**Offspring from first cross.**				
♀ 7 x ♂ 4	WT/WT/WT	♂	8	6
♀ *Neil1$^{+/-}$/Neil2$^{+/+}$/Neil3$^{+/-}$* x ♂ *Neil1$^{+/-}$/Neil2$^{+/-}$/Neil3$^{+/-}$*	Het/Het/WT	♂	9	
	WT/WT/Het	♀	10	
	KO/Het/Het	♀	11	
	Het/WT/Het	♀	12	
	WT/WT/Het	♀	13	

Neil1$^{+/+}$/Neil2$^{+/+}$/Neil3$^{-/-}$* x *Neil1$^{-/-}$/Neil2$^{+/-}$/Neil3$^{+/+}$

	Expected %	Observed	Observed %
Het/WT/Het	50	4/7	57
Het/Het /Het	50	3/7	43

Neil1$^{+/-}$/Neil2$^{+/+}$/Neil3$^{+/-}$* x *Neil1$^{+/-}$/Neil2$^{+/-}$/Neil3$^{+/-}$

	Expected %	Observed	Observed %
WT/WT/WT	3.1	1/6	
WT/WT/Het	6.3	2/6	
HetWT/WT	6.3		
Het/WT/Het	12.5	1/6	
WT/WT/KO	3.1		
Het/WT/KO	6.3		
WT/Het/WT	3.1		
WT/Het//Het	6.3		
Het/Het/WT	6.3	1/6	
Het/Het/Het	12.5		
WT/Het/KO	3.1		
Het/Het/KO	6.3		
KO/WT/WT	3.1		
KO/WT/Het	6.3		
KO/WT/KO	3.1		
KO/Het/WT	3.1		
KO/Het/Het	6.3	1/6	
KO/Het/KO	3.1		

♀ 6 x ♂ 1	**KO/KO/KO**	♂	14	7
♀ *Neil1*$^{+/-}$/*Neil2*$^{+/-}$/*Neil3*$^{+/-}$ x ♂ *Neil1*$^{+/-}$/*Neil2*$^{+/-}$/*Neil3*$^{+/-}$	Het/Het/WT	♂	15	
	Het/WT/KO	♂	16	
	KO/KO/Het	♀	17	
	Het/Het/Het	♀	18	
	WT/WT/Het	♀	19	
	WT/WT/Het	♀	20	

***Neil1*$^{+/-}$/*Neil2*$^{+/-}$/*Neil3*$^{+/-}$ x *Neil1*$^{+/-}$/*Neil2*$^{+/-}$/*Neil3*$^{+/-}$**

	Expected %	Observed	Observed %
WT/WT/WT	1.6		
WT/WT/Het	3.1	2/7	
WT/Het/WT	3.1		
WT/Het/Het	6.3		
Het/WT/WT	3.1		
Het/WT/Het	6.3		
Het/Het/WT	6.3	1/7	
Het/Het/Het	12.5	1/7	
WT/WT/KO	1.6		
WT/Het/KO	3.1		
Het/WT/KO	3.1	1/7	
Het/Het/KO	6.3		
WT/KO/WT	1.6		
WT/KO/Het	3.1		
Het/KO/WT	3.1		
Het/KO/Het	6.3		
WT/KO/KO	1.6		
Het/KO/KO	1.6		
KO/WT/WT	1.6		
KO/WT/Het	3.1		
KO/Het/WT	3.1		
KO/Het/Het	6.3		
KO/WT/KO	1.6		
KO/Het/KO	3.1		
KO/KO/WT	1.6		
KO/KO/Het	3.1	1/7	
KO/KO/KO	1.6	1/7	

**Pairings: Offspring from second cross.**				
♀ 11 x ♂ 14	KO/Het/KO	♂	21	7
♀ *Neil1$^{-/-}$/Neil2$^{+/-}$/Neil3$^{+/-}$* x ♂ *Neil1$^{-/-}$/Neil2$^{-/-}$/Neil3$^{-/-}$*	KO/KO/Het	♂	22	
	KO/Het/Het	♂	23	
	KO/KO/Het	♂	24	
	KO/KO/Het	♀	25	
	KO/KO/Het	♀	26	
	KO/Het/KO	♀	27	
♀ 17 x ♂ 14	**KO/KO/KO**	♀	28	6
♀ *Neil1$^{-/-}$/Neil2$^{-/-}$/Neil3$^{+/-}$* x ♂ *Neil1$^{-/-}$/Neil2$^{-/-}$/Neil3$^{-/-}$*	KO/KO/Het	♀	29	
	**KO/KO/KO**	♀	30	
	KO/ KO/Het	♂	31	
	**KO/KO/KO**	♂	32	
	KO/KO/Het	♂	33	
**Pairings: Offspring from second and third cross.**				
♀ 25 x ♂ 14	KO/KO/Het	♀	34	3
♀ *Neil1$^{-/-}$/Neil2$^{-/-}$/Neil3$^{+/-}$* x ♂ *Neil1$^{-/-}$/Neil2$^{-/-}$/Neil3$^{-/-}$*	KO/KO/Het	♀	35	
	KO/KO/Het	♂	36	
♀ 29 x ♂ 14	**KO/KO/KO**	♀	37	7
♀ *Neil1$^{-/-}$/Neil2$^{-/-}$/Neil3$^{+/-}$* x ♂ *Neil1$^{-/-}$/Neil2$^{-/-}$/Neil3$^{-/-}$*	KO/KO/Het	♀	38	
	KO/KO/Het	♀	39	
	**KO/KO/KO**	♀	40	
	**KO/KO/KO**	♂	41	
	KO/KO/Het	♂	42	
	KO/KO/Het	♂	43	
♀ 29 x ♂ 32	KO/KO/Het	♂	54	10
♀ *Neil1$^{-/-}$/Neil2$^{-/-}$/Neil3$^{+/-}$* x ♂ *Neil1$^{-/-}$/Neil2$^{-/-}$/Neil3$^{-/-}$*	KO/KO/Het	♂	55	
	**KO/KO/KO**	♂	56	
	**KO/KO/KO**	♀	57	
	**KO/KO/KO**	♀	58	
	KO/KO/Het	♀	59	
	**KO/KO/KO**	♀	60	
	KO/KO/Het	♀	61	
	**KO/KO/KO**	♀	62	
	KO/KO/Het	♀	63	

Neil1$^{-/-}$/Neil2$^{+/-}$/Neil3$^{+/-}$* x *Neil1$^{-/-}$/Neil2$^{-/-}$/Neil3$^{-/-}$

	Expected %	Observed	Observed %
KO/Het/Het	25	1/7	
KO/Het/KO	25	2/7	
KO/KO/Het	25	4/7	
KO/KO/KO	25		

Neil1$^{-/-}$/Neil2$^{-/-}$/Neil3$^{+/-}$* x *Neil1$^{-/-}$/Neil2$^{-/-}$/Neil3$^{-/-}$

	Expected %	Observed	Observed %
KO/KO/Het	50	15/26	58
KO/KO/KO	50	11/26	42

Pairings: *Neil1$^{-/-}$/Neil2$^{-/-}$/Neil3$^{-/-}$ x Neil1$^{-/-}$/Neil2$^{-/-}$/Neil3$^{-/-}$*

**Litter size**	♀	♂
7	6	1
10	6	4
9	4	5
9	4	5
8	3	5
6	2	4
8	3	5
9	5	4
10	6	4
9	5	4
9	5	4
9	6	3

**Genotype**	**Sex**	**Total number**
**KO/KO/KO**	♀	**54**
**KO/KO/KO**	♂	**48**
